# Supplementary material for: A UMLS-based spell checker for natural language processing in vaccine safety
Source: BMC Med Inform Decis Mak. 2007 Feb 12;7:3. doi: 10.1186/1472-6947-7-3 (PMC1805499; doi:10.1186/1472-6947-7-3)
Supplement: Additional file 13 — RAP application directory. Sets up directory for RAP (RDF application for PHP) source code [file 1472-6947-7-3-S13.gz › rap/doc/tutorial/vocabularies.htm]

Tutorial: Using Common Vocabularies


### RDF API for PHP V0.9.1

# Tutorial: Using Common Vocabularies

This turorial is part of the RAP - Rdf API for PHP documentation.

RAP provides build-in support for the terms of a range of commonly known vocabularies. With version 0.9.1, RAP directly supports the following 8 vocabularies:

|  |  |  |
| --- | --- | --- |
| **Vocabulary** | **Website** | **Specification** |
| RDF | http://www.w3.org/RDF/ | http://www.w3.org/RDF/#specs |
| RDFS | http://www.w3.org/TR/rdf-schema/ | http://www.w3.org/TR/rdf-schema/ |
| OWL | http://www.w3.org/TR/owl-ref/ | http://www.w3.org/TR/owl-ref/ |
| Dublin Core (DC) | http://dublincore.org/ | http://dublincore.org/documents/dcmi-terms/  http://dublincore.org/documents/dces/ |
| Friend of a Friend (FOAF) | http://www.foaf-project.org/ | http://xmlns.com/foaf/0.1/ |
| RSS | http://web.resource.org/rss/1.0/ | http://web.resource.org/rss/1.0/spec |
| ATOM | http://semtext.org/atom/index.html | http://www.atomenabled.org/developers/syndication/atom-format-spec.php |
| VCARD | http://www.w3.org/TR/vcard-rdf | http://www.w3.org/TR/vcard-rdf#3 |

This tutorial describes the usage of terms from these vocabularies within RAP.

### Contents:

|  |  |
| --- | --- |
| 1. | Including the Necessary Vocabulary Package |
| 2. | Using the Vocabulary |

### 1. Including the Necessary Vocabulary Package

In order to use a vocabulary you have to include the
corresponding
vocabulary package. The name of the file which you have to include depends on whether you are working on the RAP model layer model layer or on the RAP resource layer.

When you want to use FOAF together with a `MemModel` you have to include:

`include( RDFAPI_INCLUDE_DIR . 'vocabulary/FOAF_C.php');`

In case of working with a `ResModel` you have to include:

`include( RDFAPI_INCLUDE_DIR . 'vocabulary/FOAF_RES.php');`

The following table lists all include packages for the different vocabularies:

|  |  |  |
| --- | --- | --- |
| **Vocabulary** | **Together with MemModel** | **Together with ResModel** |
| RDF | `vocabulary/RDF_C.php` | `vocabulary/RDF_RES.php` |
| RDFS | `vocabulary/RDFS_C.php` | `vocabulary/RDFS_RES.php` |
| OWL | `vocabulary/OWL_C.php` | `vocabulary/OWL_RES.php` |
| Dublin Core (DC) | `vocabulary/DC_C.php` | `vocabulary/DC_RES.php` |
| Friend of a Friend (FOAF) | `vocabulary/FOAF_C.php` | `vocabulary/FOAF_RES.php` |
| RSS | `vocabulary/RSS_C.php` | `vocabulary/RSS_RES.php` |
| ATOM | `vocabulary/ATOM_C.php` | `vocabulary/ATOM_RES.php` |
| VCARD | `vocabulary/VCARD_C.php` | `vocabulary/VCARD_RES.php` |

### 2. Using the Vocabulary

Now you are able to create resources for vocabulary terms by calling one simple method instead of generating them with `$resouce = new Resource('uri')`. To create a RDFS- comment for example you have to call:

`$comment = RDFS::COMMENT();`

instead of:

`$comment = new Resource(http://www.w3.org/2000/01/rdf-schema#comment);`

If you included the `PACKAGE_VOCABULARY_CLASS` package $comment is a Resource with the URI
http://www.w3.org/2000/01/rdf-schema#comment. If you included the `PACKAGE_VOCABULARY_RES` package $comment is a ResResource with the same URI.

Normally the method to create a term is named like the term in the vocabulary using upper case. There are some reserved words in PHP like 'class' or 'list' so the methods to create these resources have other identifiers. To generate an OWL Class (http://www.w3.org/2002/07/owl#Class) for example use the method `OWL::OWL_CLASS()`. To find out how these methods are named have a look at the corresponding vocabulary file.

### 3. Backward Compatibility

For backward
compatibility
reasons, we are still supporting RAP 0.8's `PACKAGE_VOCABULARY` package. Including this package will create MemModel resources for all terms in all vocabularies. This is very memory consuming and should thus be avoided.

In order to use the old vocabularies you have to include:

`include( RDFAPI_INCLUDE_DIR . PACKAGE_VOCABULARY);`
